# Evaluation of protective factors in caries free preschool children: a case-control study

**DOI:** 10.1186/s12903-020-01154-y

**Published:** 2020-06-26

**Authors:** Reza Yazdani, Simin Zahra Mohebbi, Maryam Fazli, Maryam Peighoun

**Affiliations:** Community Oral Health Department, School of Dentistry, Tehran University of Medical Sciences, Tehran, Iran

**Keywords:** Caries free, Children, ECC, Protective factors, Case-control study

## Abstract

**Background:**

Increasing the proportion of caries-free children following the WHO’s global target has led to more desirable welfare and a higher level of quality of life for children. In the present study, we aimed to evaluate the factors contributing to a caries-free condition in preschool children as a basic action towards the global goals of children’s oral health.

**Methods:**

This was a case-control study evaluating the protective factors contributing to dental caries free in 4–6-year-old children in Tehran/Iran in 2017. 500 preschool children and their mothers were selected from 22 randomly selected preschools and were enrolled in the study. The participants were divided into two case (caries-free) and control (with dental caries) groups. The data were collected using two data gathering tools; the child oral examination form and the mother’s valid questionnaire. The latter included three domains; socio-demographic factors, behavioral oral health measures, and feeding practices and dietary habits. The criteria for caries detection were cavities in the enamel and dentine. A logistic regression model was applied to identify caries-free protective factors (*P* < 0.05).

**Results:**

Among 230 caries-free and 270 non-caries-free children who participated in the study, boys were more caries-free (*P* = 0.001). The protective factors against dental caries that were identified in the study were dental check-up as the cause of dental visit, being the first child in the family, the fewer sessions night feeding of the child’s, family’s house ownership, and parent’s university education (*P* < 0.05).

**Conclusions:**

Dental health can be achieved by considering protective factors like the regular dental check-up and socio-economic factors. Communities are invited to pay close attention to these important protective factors as far as they can increase the proportion of caries-free among preschool children especially in countries with developing oral health care systems.

## Background

Oral health activities have shifted toward improving the proportion of children without dental caries worldwide [1]. Caries-free children eat, drink, and chew well enough to have a desirable lifestyle and better health. Children with untreated and/or neglected dental caries are prone to complications such as pain, missed school days, compromised eating habits, low self-esteem, and inappropriate nutritional patterns, which may consequently impair their physical development, well-being, and oral health-related quality of life [2]. In low-income countries, most of dental caries are left untreated. Teeth affected by caries are often extracted when they cause pain or discomfort [3]. The estimated treatment costs and the expertise required for the treatment of the disease put pressure on the economy, which makes it necessary to trace the contributing protective factors in caries-free children in order to tailor a preventive economic approach to act against the early childhood caries as a deep-rooted disease [3]. The esthetic issue should also be emphasized, which may affect social interaction and prevent social withdrawal and shyness [4].

The early childhood caries (ECC) are on the other side of the dental health spectrum in children, which is a major global public health concern and the most widespread non-communicable disease. It is also the most prevalent condition included in the 2015 Global Burden of Disease Study, ranking 12th for deciduous teeth (affecting 560 million children) [5]. Data from the United States of America show a high prevalence of ECC with 40% of children acquiring caries by kindergarten age, while 12% of 3-year-old children in the United Kingdom have ECC [6]. The prevalence of ECC is estimated as 20–80% by the report of the WHO Regional Office for the Eastern Mediterranean region [7]. In Iran, a national survey of 6 year-old-children in 2016 showed that only 14.2% of the children in Tehran and 8.9% of the children in Iran were caries-free [8].

The percentage of caries-free children does not meet the target established by the WHO/FDI for 2010, which indicates that 90% of children should be caries-free at ages 5 and 6 years. It did not even meet the WHO target for 2000 [8, 9]. It is important to note that studies must be compared with caution because there are variations in the diagnostic criteria and indices used to evaluate the disease in this age group. Important factors such as socioeconomic status, parents’ habits and knowledge of oral health affect children’s oral health status [10, 11]. It is well established that children living in families with low income and low educational attainment have poorer oral health and access to dental care than children with more affluent and educated families [12]. As shown in a systematic review, almost 90 risk factors have been described for ECC [10]. This complex etiology of dental caries has intrigued researchers to dig deeper into the various protective factors, which could be involved in avoiding the ignorance of the multifactorial nature of dental caries in every society and also to recognize the preventive aspects of this disease toward dental caries in children. Therefore, to perform our study, we tried to emphasize all factors that directly or indirectly affect the teeth to be free of caries. Many studies have investigated the factors associated with dental caries, but few have evaluated the protective factors. The present study was conducted to identify and evaluate the factors contributing to a caries-free condition in children aged 4–6 years.

## Methods

### Study design, sample size, and data collection

This was a case-control study evaluating the protective factors of dental caries-free in 528, 4–6-year-old children and their mothers selected from 22 randomly selected preschools that were enrolled in the study from April to December 2017 in Tehran/ Iran. The study was approved by the Ethics Committee of Tehran University of Medical Sciences (IR.TUMS.REC.1395.6070). The sample size was calculated using the EPI tools epidemiological calculator for case-control studies with a confidence level of 95%, and power of 0.8, and an equal number of cases and controls. For preschool selection, which could represent the whole city, the megacity of Tehran was divided into 22 strata according to the divisions of Tehran Municipality and the Ministry of Education. We actually applied the multi-stage cluster random sampling for preschool random selection. In fact, municipality districts were the stratum, and preschools in each stratum were clusters. We got a list of preschools in each district of Tehran Education Organization, and subsequently, one preschool (one cluster) was randomly selected in each stratum as a primary sampling unit and in each preschool, normal healthy 4–6-year-old children were included in the study by an oral examination. The healthy children were selected based on the children’s health report issued by a physician, which is accessible in children’s health files in the preschool office. The child would be placed in the case group if s/he was caries-free and would be placed in the control group if s/he had dental caries after obtaining informed consent from her/his mother. They were actually friends and neighbors controls. It was important to recruit 12 caries-free children and 12 controls in each preschool [13]. As we did not have enough cases in three preschools, we chose more controls.

In the next step, the mothers who were invited to the preschools were approached and the purposes of the study and interview were clearly explained to them and they were assured of the lack of side effects. After obtaining their approval for participation, they were requested to sign an informed consent form prior to enrollment. 27 mothers and their children were not willing to participate in the study; one mother and her child immigrated to another city, so the final sample size was 500 mothers and their preschool children. All mothers were motivated by offering preventive procedures such as professional dental cleaning, and educational sessions on proper oral hygiene measures, and the required dental treatment after the study.

The study was divided into two stages; stage 1 included the intra-oral examination of the children, and stage 2 was a mother questionnaire survey.

### Children intra-oral examination

In stage 1, the children’s teeth were examined by an experienced and calibrated dentist (MP). The intra-rater reliability by Cohen’s kappa coefficient was 0.89 as an almost perfect agreement. The intraoral examination was conducted in accordance with the WHO standards, in an area well-lit with natural and headlamp using disposable plain dental mirrors, gauze wipes, and wooden tongue depressors [13]. The criteria for caries detection were cavities in the enamel and dentine. The children were examined in the sitting position on a chair in the preschool facing the window by the dentist. The children were divided into two groups. Participants in group 1 (cases) were caries-free children, which means that they did not have any decayed, missing, or filled primary teeth. A child was considered to be suffering from ECC when (s) he complied with the definition of the American Academy of Pediatric Dentistry (AAPD) defining ECC as the presence of one or more decayed, missing, or filled tooth surface in any primary tooth in a child of 71 months of age or younger [14]. These children who had AAPD criteria were categorized as group 2 (controls). As the cases and their controls were almost selected from the same preschool, they were approximately similar from the points of the calendar time of examination, and allocation, area of living, and overall health.

### Mothers’ questionnaire survey

In stage 2, the mothers were interviewed by another dentist who was unaware of the results of the children’s oral examination performed by the first dentist. A standard and valid structured questionnaire was considered for the interviews [15]. The questionnaire was evaluated for content validity once more by a group of five experts of community oral health and epidemiology. The questions that were found to be confusing were revised and retested on 10 mothers to check the questionnaire’s stability. The questionnaire reliability was determined by the internal consistency of 0.80 based on Cronbach’s alpha coefficient. Temporal consistency was evaluated by the test- retest method for categorical variables by kappa of 0.84.

The responses were recorded in face-to-face interviews. In such a study the level of participants’ education might be different. Therefore, our method in completing the questionnaire was the interview for two reasons. The first reason was to improve the accuracy of answers and the second reason was to prevent missed data.

The questionnaire included three domains, sociodemographic factors, behavioral oral health measures, and feeding practices and dietary habits. Sociodemographic data included sex, age, the number of siblings, and the parents’ level of education.

Bangkok Declaration of the International Association of Peadiatrics Dentistry (IAPD) on Early Childhood Caries emphasizes that dental caries is determined by biological, behavioral, and psychosocial factors [16]. “ECC is a global dilemma, and with the exception of a multitude of risk factors like oral hygiene maintenance, its incidence is associated with additional factors, such as socioeconomic and education status” [17]**.** As it was established that education is an important socioeconomic variable in health-related studies, maternal and paternal education were originally assessed using seven categories that were there after dichotomized into < 12 vs. 12+ years of schooling or respectively non-university education and university education since these groups contained most participants.

In behavioral oral health measures, the mothers were asked about the frequency of dental brushing in their children. The answers were categorized as “no brushing and equal to or less than once/day”; these were children who brushed their teeth less than 2 times/day, and “more than once/day”; these included children who brushed their teeth 2 times/day [18]. They were specifically asked if brushing was performed at night or at a random time. They were asked if brushing was self-implemented or performed by the mother or others. Finally, the mothers were asked about any previous dental visit, whether it was early (at the child’s age of 1 year and before it) or late (after the child’s age of 1 year), and its cause.

We did not have a question about toothpaste containing fluoride because even if 4–6 year- old children applied toothpaste with 500 ppm fluoride, it should not act as a protective factor. Studies have shown that toothpaste containing less than 1000 ppm fluoride does not have an anti-caries effect [19].

Dietary and nutritional data included questions about the child’s history of night time and on-demand feeding. Information was collected about daily consumption of foods containing sugar referred to as “sugary foods”, sweets, and drinks, as well as fruit juices (the answers were categorized as 3 times or less and more than 3 times). According to Fejerscov in the book of Dental Caries; “A moderate caries increases for the individuals receiving sugary snacks 4 times daily” [20]. On the other hand, a normal diet frequency includes three main meals and two snack meals. As our study groups were children who need more meals and snacks that are usually sugary, so we decided to choose three times daily consumption as our cut of point.

Another question that mothers were asked about was related to Iron drop consumption for which mothers would answer “yes” or “no”. This question was actually included to clarify mothers’ misunderstanding about the Iron effect on dental caries that happens most of the time.

### Statistical methods

Both the questionnaire and oral examination forms were manually checked for the completion of the required information.

The collected data were entered into the SPSS software version 21 for statistical analysis (IBM Corp. Released 2012. IBM SPSS Statistics for Windows, Version 21.0. Armonk, NY: IBM Corp). Before starting the study, and during the stage of study design, we weighted all preschools from the point of the target populations in all districts by categorization of preschools on their population/area (density). We randomly chose one preschool from the same density preschools in each district. At the end, the sample included the weighted children.

We performed case-control pairing appropriately to avoid the effects of a confounding variable in sampling and, moreover we adjusted for potential confounders through mathematical modeling using regression method (logistic regression) in the analysis step.

Chi-square test was used to find significant differences in categorical variables between the two groups. If the differences were significant, we included that variable in multiple logistic regression. A standard logistic regression model was used to identify the determinants of being caries-free in children by considering independent variables simultaneously. The final model was presented with a backward technique. Odds ratios were calculated for all possible factors associated with the prevalence of being caries-free. *P* values< 0.05 were considered as statistically significant. Since the less sugar consumption are one of the most important dietary factor in the development of the less dental caries, despite the removal of sugar consumption, we again included sugar consumption to the final regression model and maintained it in the model.

## Results

The background descriptive and analytical data of this case-control study are demonstrated in Table 1 and Tables 2, 3 and 4 respectively. The cases were 230 children who were caries-free and the controls were 270 children who had dental caries.

**Table 1:** Socio- demographic characteristics of study participant regards to compare between caries free and non caries free children

Variables	Cases n:230	Controls n:270	95%CI
**Gender**			
Male	145 (52.3%)	132 (47.7%)	
Female	83 (37.9%)	136 (62.1%)	1.26_2.58
**Primogenitor**			
No	38 (24.4%)	118 (76.6%)	2.51_5.99
Yes	192 (55.8%)	152 (44.2%)	
**Who cares of child**			
Mother	189 (47.7%)	207 (52.3%)	0.90_2.18
Others	41 (39.4%)	63 (60.6%)	
**Age of care giver**			
> 35	26 (34.7%)	49 (65.3%)	1.05_2.93
≤ 35	204 (48.2%)	219(%52.3%)	
**House ownership**			
No	99 (38.2)	160 (61.8)	0.36_0.73
Yes	130 (54.9%)	107 (45.1%)	
**Family child size**			
> 1child	42 (24.6%)	129 (75.4%)	2.66_6.11
1 child	186 (56.6%)	141 (43.1%)	
**Mother’s educational level**			
Non university edu	45 (19.8%)	182 (80.2%)	0.08_0.18
University edu	185 (67.8%)	88 (32.2%)	
**Father’s educational level**			
Non university edu	19 (14.9%)	111 (85.4%)	0.08_0.22
University edu	211 (57.0%)	159 (43.0%)

**Table 2:** Non adjusted caries free protective factors according to background and socio- demographic variables in two case and control 4–6 year- old children groups (*n = 500) by logistic model

Variables	Cases n:230	Controls n:270	OR	95%CI	***p***-Value
**Gender**					
Male	145 (52.3%)	132 (47.7%)	Ref. 1.80	1.26–2.58	0.
Female	83 (37.9%)	136 (62.1%)			
**Primogenitor**					
No	38 (24.4%)	118 (76.6%)	Ref.	2.51–5.99	< 0.001
Yes	192 (55.8%)	152 (44.2%)	3.92		
**Who cares of child**					
Mother	189 (47.7%)	207 (52.3%)	Ref. 1.40	0.90–2.18	0.13
Others	41 (39.4%)	63 (60.6%)			
**Age of care giver**					
> 35	26 (34.7%)	49 (65.3%)	Ref. 1.76	1.05–2.93	0.03
≤ 35	204 (48.2%)	219(%52.3%)			
**House ownership**					
No	99 (38.2)	160 (61.8)	Ref. 0.51	0.36–0.73	< 0.001
Yes	130 (54.9%)	107 (45.1%)			
**Family child size**					
> 1child	42 (24.6%)	129 (75.4%)	Ref. 4.05	2.66–6.11	< 0.001
1 child	186 (56.6%)	141 (43.1%)			
**Mother’s educational level**					
Non university edu	45 (19.8%)	182 (80.2%)	Ref. 0.12	0.08–0.18	< 0.001
University edu	185 (67.8%)	88 (32.2%)			
**Father’s educational level**					
Non university edu	19 (14.9%)	111 (85.4%)	Ref. 0.13	0.08–0.22	< 0.001
University edu	211 (57.0%)	159 (43.0%)			

Ref.odds ratio references

**Table 3:** Non adjusted caries free protective factors according to each behavioral variables in two case and control 4–6 year- old children groups (*n = 500) by logistic model

Variables	Cases n:230	Controls n:270	OR	95%CI	***p***-Value
**Antibiotic consumption**					
No	101 (47.4%)	112 (52.9%)	Ref. 1.11	0.77–1.58	0.58
Yes	129 (44.9%)	158 (55.1%)			
**Iron drop consumption**					
Yes	226 (47.2%)	253 (52.8%)	Ref. 3.80	1.26–11.45	0.01
No	4 (19.0%)	17 (81.0%)			
**Water drink after Iron use**					
Yes	198 (55.0%)	162 (45.0%)	Ref. 4.13	2.64–6.44	< 0.001
No	32 (22.9%)	108 (77.1%)			
**Mother doesn’t know how to brush child’s teeth**					
Agree	17 (15.5%)	93 (84.5%)	Ref. 0.15	0.09–0.26	< 0.001
Disagree	210 (55.3%)	170 (44.7%)			
**Mother doesn’t have time to brush child’s teeth**					
Agree	15 (15.5%)	82 (84.5%)	Ref. 0.16	0.09–0.28	< 0.001
Disagree	215 (53.8%)	170 (46.3%)			
**Mother isn’t able made the child to brush**					
Agree	110 (44.4%)	138 (55.6%)	Ref. 0.85	0.60–1.21	0.37
Disagree	120 (48.4%)	128 (51.6%)			
**Duration of breast feeding**					
≥ 6 months	161 (53.5%)	140 (46.5%)	Ref. 2.15	1.49–3.11	< 0.001
< 6 months	69 (34.8%)	129 (65.2%)			
**Age of bottle feeding start**					
≥ 6 months	191 (48.9%)	202 (51.4%)	Ref. 1.60	1.03–2.50	0.04
< 6 months	39 (37.1%)	66 (62.9%)			
**Milk feeding during sleep**					
Yes	179 (41.4%)	253 (58.6%)	Ref. 0.24	0.13–0.42	< 0.001
No	51 (75.0%)	17 (25.0%)			
**Frequency of night feeding**					
> 1 time	81 (31.5%)	176 (68.5%)	Ref. 3.44	2.38–4.98	< 0.001
≤ 1 time	149 (61.3%)	94 (38.7%)			
**‘Frequency of sugar consumption/day**					
≥ 3 times	5 (8.5%)	54 (91.5%)	Ref. 0.09	0.04–0.23	< 0.001
< 3 times	225 (51.0%)	216 (49.0%)			
**Dental cleaning appliance**					
Others	41 (22.0%)	145 (78.0%)	Ref. 0.19	0.12–0.28	< 0.001
Brush	189 (60.2%)	125 (39.8%)			
**Who brushes child’s teeth**					
Child	89 (56.3%)	69 (43.7%)	Ref. 1.84	1.26–2.69	0.002
Others	141 (41.2%)	201 (58.8%)			
**Frequency of brushing/ day in child**					
≤ 1 time/day	127 (32.9%)	259 (67.1%)	Ref. 19.10	9.90–36.84	< 0.001
> 1 time/day	103 (90.4%)	11 (9.6%)			
**Frequency of brushing/ day in parents**					
≤ 1 time/day	72 (28.0%)	185 (72.0%)	Ref. 4.78	3.27–6.98	< 0.001
> 1 time/day	158 (65.0%)	85 (35.0%)			
**Child’s age at tooth paste starting**					
> 3 years old	61 (35.9%)	109 (64.1%)	Ref. 0.53	0.36–0.78	0.001
≤ 3 years old	169 (51.2%)	161 (48.8%)			
**Cause of dental visit**					
Others	7 (3.5%)	193 (96.5%)	Ref. 79.85	35.97–177.24	< 0.001
Dental check-up	223 (74.3%)	77 (25.7%)			
**First dental visit**					
< 1 year old	39 (65.0%)	21 (35.0%)	Ref. 2.42	1.38–4.25	0.002
≥ 1 year old	191 (43.4%)	249 (55.6%)			
**Routine dental visit**					
No	35 (19.1%)	148 (80.9%)	Ref. 6.86	4.45–10.58	< 0.001
Yes	193 (61.9%	119 (38.1%)			

Ref:.odds ratio references

**Table 4:** Adjusted caries free protective factors according to variables in two case and control 4–6 year old children groups (*n = 500) by multiple logistic model

Protective Factors		Odds Ratio	***p***-Value	95%CI For Odds Ratio
**Primogenitor**				
No		Ref. 3.90	< 0.001	1.85–8.22
*Yes*				
**House ownership**				
*No*		Ref. 0.38	0.006	0.19–0.76
*Yes*				
**Mother’s educational level**				
Non university edu University edu		Ref. 0.29	0.002	0.13–0.63
**Father’s educational level**				
Non university edu		Ref.0.39	0.03	0.16–0.94
University edu				
**Number of feeding during night**				
> 1 time/day		Ref. 2.05	0.05	1.01–4.17
≤ 1time/day				
**Frequency of sugar**	≥3times	Ref. 0.47	0.34	0.12–2.11
**consumption/day**	< 3 times			
**Cause of dental Visit**	Others Check-up	Ref. 87.22	< 0.001	36.18–238.52

Table 2 shows unadjusted protective factors of caries-free in the background and socio-economic variables. According to Table 2, 52% of all boys participated in the study who were caries-free belonging to the case group. Of all girls who participated in the study only 38% were in the caries-free group. At first glance, as the chance of being caries-free were1.80 times higher in girls compared with boys, sex could be a protective factor. Of all who were the first child of the family, 55.8% were in the case group. The chance of being caries free was 3.92 times more in those who were the first child in the family.

Children whose parents did not own a house had a lower chance of being caries-free (OR: 0.51, CI: 0.36–0.73). There were statistically significant differences in the background and sociodemographic variables between the two groups of case and control children (Table 2).

Table 3 indicates the chance of being caries-free in behavioral protective factors in the unadjusted model. There were statistically significant differences in all behavioral variables between the cases and controls except in two variables: antibiotic consumption and mothers who were not able to make their children brush their teeth (*P* = 0.58 and *P* = 0.37, respectively).

Table 3 also shows the chance of being caries-free was 19 times higher in children who brushed their teeth more than once per day compared with children who brushed their teeth one or less than one a day (*P* < 0.001). It was also true in children whose parents had similar dental brushing behavior (OR: 4.78, CI: 3.27–6.98). Furthermore, children who drank water after iron consumption had the frequency of night feeding ≤1 time, and had routine dental visits had increased chance of being caries-free by 4.13, 3.44, and 6.86 times, respectively. The chance of dental health was 1.84 times higher in children whom their caregivers were responsible for children’s oral hygiene practice (*P* = 0.002).

Table 4 demonstrates the results of forward stepwise logistic regression analysis, indicating the final or master caries-free protective factors model. Among dental visit causes, dental check-up posed a greater chance of (80.64 times more than the other causes of dental visit) being a protective factor in caries-free children. Variables included, “to be a primogenitor or first child in the family” (OR: 3.56, CI: 1.75–7.25), “the less number of feeding during the night “(OR: 2.0, CI: 1.09-3.97), “the family house ownership” (OR: 0.45, CI: 024–0.86), “the father’s higher educational level” (OR: 0.32, CI: 0.13–0.74),” the mother’s higher educational level” (OR: 0.26, CI: 0.12–0.56) were the next five ranked variables in this model, respectively. Although sugar consumption was removed in the adjusted model, we maintained it because of the proven effect of the less sugary diet on dental health. The differences between every adjusted and unadjusted odds ratio showed the confounding effect of other variables.

## Discussion

In this study, the protective factors against dental caries were emphasized instead of the risk factors. The findings of this health-oriented study were almost consistent with the results of other studies but on the health side of the disease spectrum [11].

Interestingly, the number of siblings in the family was one of the significant protective factors in the present study; the fewer siblings a child had, the greater his or her chance to be caries-free. One explanation for this finding may be that the parents’ attention towards their children’s oral health is shared or divided between a numbers of children or the oral health may be neglected; therefore in small families, the children receive the necessary care, resulting in an increased chance of being caries-free. Horizontal transmission of dental caries among siblings may also play a role in this regard. This finding was in agreement with the results of other studies [21, 22].

Studies show that the parents’ level of education had an association with the status of dental caries in children. The result of the present study, which evaluated the parent’s higher education effect on children’s caries-free were similar to the results of another study that attributed dental caries to the lack of information about oral health care in children and reported a reverse correlation between dental caries in children and mothers’ and fathers’ educational level [23]. In the present study, the mothers’ and fathers’ university education had a health-oriented direction effect on caries-free in comparison with ECC studies [21]. We also emphasized the role of paternal university education that was pointed to in fewer studies.

One of our findings in the present study was more liability to be caries-free among children with a routine dental visit. Children in the case group had more dental check-up as the cause of the dental visit. We found a strong association between dental visits and caries-free status. This finding was reversely underscored in several studies on ECC [11, 15, 18–21]. The wide confidence interval of this finding as a study limitation may be because a small proportion of the reasons for dental visit that belonged to other reasons like pain in the caries-free group. Table 2 shows that 74.3% of the cause of the dental visit in the case group was dental check-up while 96.5% of the causes of dental visits in the control group were other reasons.

In this study, similar to the results of most other studies on dental caries risk factors [21]; more frequency of night feeding in children decreased the chance of being caries-free, of course in health-oriented directions, “the less night feeding, and the more dental health”.

Many studies have reported that the frequency of sugary foods intake is higher in children with dental caries than those who are caries-free, which is consistent with the results of several investigations indicating that the incidence of dental caries increases when sugar-containing snacks are consumed more than four times a day [10]. However, in the present study, the chance of being caries-free decreased when sugary foods were consumed more than three times a day. On the contrary, in another study, the researchers state that “consumption of sugar-added beverages and sweets is not statistically and significantly associated with dental caries” [24]. It should not be forgotten that other factors like topical fluoride could play their positive simultaneous roles. Of course, this study like any other case-control studies has its own limitations, as the measurement of the disease was established after the development of the disease and as a result, it is prone to both recall and observer biases. The question regarding sugar consumption may be accompanied by these systematic errors. It is possible that mothers forgot the exact frequency of feeding sessions or sugary food intake per day. The low sugar consumption, which was assessed in this study, was not associated with caries-free. This might be resulted from measurement bias, so we hope to use a validated questionnaire to measure sugar consumption in our future studies.

Regarding the association between the Iron drop and caries-free status, it should be added that Iron drops are distributed freely to all children in Iran through the Primary Health Care System. As shown in Table 1 almost all children consumed Iron drop in both case and control groups. As we expected there was a significant association between Iron drop consumption and caries-free status. It means that Iron drop consumption is not responsible for dental caries. We suggest performing educational meetings to clarify this issue for mothers.

Most studies have shown that ECC-free groups adhere better to proper oral hygiene practices, especially the frequency of brushing, regular brushing at night, as well as the supervised oral hygiene measures by the caregiver instead of the child him (her) self [21].

Our study found dental brushing frequency as a protective factor in dental caries prevention. Despite the better chance of dental brushers in comparison with less or no dental brushers in being caries-free, the wide confidence seems to be a study limitation. On careful consideration, we find out that it may be because of the few numbers of children with dental brushing behavior of more than 1 time per day in the control group.

The use of fluoride toothpaste has been proven to be associated with caries reduction [20, 25]. Regarding this topic, we only asked about the age that the child started fluoride-containing toothpaste. It should be added that the oral care system in our country and also national toothpaste manufacturers present 500 ppm fluoride-containing toothpaste to 3 to 6-year-old children. There was a lack of information regarding the type of toothpaste (fluoride toothpaste or non-fluoride toothpaste) and the amount of fluoride in the toothpaste in this study. Although this could be one of our limitations at first glance, there was a logic behind that as in many countries like Iran that 4–6-year-old children still receive 500 ppm fluoride in their toothpaste, it makes no difference asking whether or not to they applied 500 ppm fluoride because this amount of fluoride cannot act as a protective factor. Studies have already shown that toothpaste containing less than 1000 ppm fluoride does not have an anti-caries effect [19]. But we admit that we did not evaluate the other sources of fluoride and whether parents used toothpaste containing more than 500 ppm fluoride for children. Also, the current unreal propaganda against fluoride might hinder parents to use fluoride-containing toothpaste for their children, which was not assessed in this study either. We could call them as our study limitations. Of course, we should differentiate between dental brushing and the effects of fluoride in toothpaste that cannot logically be achieved in case-control studies.

The mothers, who play an important role in the children’s oral health behaviors, should be informed that their dental health habits influence their children’s oral health, and consequently their quality of life [26]. We also emphasized the mothers’/caregivers’ role in the child’s oral health in the present study.

Dental caries is related to socioeconomic factors. The present study showed that owning a house, which was more in cases than controls, had an association with a lack of dental caries. This finding is in line with the results of a study that reported a significant correlation between owning a house and a lack of dental caries even in the low socioeconomic class [25]. The reason for this association may be the fact that low-income families have marital, social, and financial disadvantages that compromise their ability to take care of themselves and their children. These factors lead to decreased resistance to different diseases, including oral diseases. Therefore, the healthy primary dentition of children is associated with parental factors, including the parents’ socio-economic status, behaviors, and attitudes related to oral health. These factors should be considered when planning preventive and educational programs for children’s oral health. It should be admitted that we focused defectively on the parental subjects in this study.

In contrast to this study that showed a lower frequency of milk feeding (bottle or breastfeeding) at night was associated with a lack of dental caries, a recent study revealed that prolonged breastfeeding at night had no association with dental caries. Prevention of dental caries should be instituted as soon as primary teeth start erupting, especially through discouraging the consumption of factory-made sugary foods/snacks [27].

The results of some studies indicate that the risk factors associated with ECC include oral hygiene acceptance, breastfeeding for more than 16 months, daily intake of sugar more than four times a day, and history of caries in parents [28, 29]. As a limitation of the present study, we did not evaluate the effect of previous caries experience in the parents. Also, we did not evaluate the effect of the type of consumed milk because most of our mothers fed their child both by breast and formulated milk at the same time.

In the present study, the chance of being caries-free was higher in children whose mothers were equal to or younger than 35 years; therefore, the younger maternal age could be a protective factor against caries. There is less attention to the mother’s or caregiver’s age as a factor in other studies.

According to the results of this study, the determinants for being caries-free in children were dental check-up as a cause of dental visit, the number of feeding sessions during night, to be primogenitor or to be the first child in the family, owning a house in the family, father’s higher education, and mother’s higher education. The use of fluoride-containing toothpaste and fluoridated water, supervised tooth brushing twice a day, educational programs, and access to primary health care providers could be the important protective factors, which have been stated in other studies [30–34]. Since the less sugar consumption is the most important dietary factor in the development of the less dental caries, we decided to maintain it in the last model. Therefore, we put it again in the regression with other variables in the last model.

We suggest immediate attention to dental caries prevention considering the protective factors against dental caries in children. It is also needed to perform robust epidemiological studies to determine the required preventive measures and services for children. Community-based programs and professional care should begin early in the first year of life, and even before birth, to build correct hygiene and feeding practices. If dental caries occur, it is important to identify the incipient lesions before they progress. Therefore, routine dental check-ups are seriously recommended to maintain oral health and avoid expensive dental treatments. This is why oral health is integrated into Primary Health Care in some countries including Iran. In this program, the extent of oral health education and mothers’ regular training build mothers’ performance and management of children’s oral health in people-centered health services. Parents with a low level of education require special attention because their children are more susceptible to dental caries and would benefit most from community preventive care.

Finally, it is clear that societies are different in terms of socio-economic factors and culture, and this study population is not an exception. Despite our efforts, we could not choose a completely representative study sample because of the reasons such as not completely covered preschools. Moreover, due to the location restriction, the external validity of the present study is not high enough to generalize the study results; although, Tehran is an example of the country’s demographic composition.

However, it is required that our national data be used along with the data of similar studies in other countries for a better understanding of the protective factors against dental caries and the improvement of children’s oral health.

## Conclusion

Children’s dental health is an achievable goal, which should be dealt with considering the protective factors against dental caries. This study revealed that “ dental check-up”, “to be the first child in the family”, “the fewer number of night feeding sessions”, “ more than one time of dental brushing per day”, “ the family’s house ownership”, “father’s higher education “, and “mother’s higher education “ were the protective factors in caries-free children aged 4–6 years in the final model of the present study. That means regular dental check-up as a cause of dental visit along with socioeconomic factors could act as protective factors in increasing the proportion of caries-free children among preschoolers especially in countries with developing oral health care systems like Iran.

## Data Availability

Datasets and analysis of the study are available in a file in the Department of Community Oral Health, Dental School of Tehran University of Medical Sciences. The data, participants’ details and study materials will not be made publicly available due to the unfinished data analysis of the second part of the study and to protect the respondents’ identity. Of course, the corresponding author provides the current study datasets and analysis on reasonable requests under the funder permission.

## Abbreviations

ECC: Early childhood caries
SPSS: Statistical Software for Social Sciences
